# Quantifying Mosaic Development: Towards an Evo-Devo Postmodern Synthesis of the Evolution of Development via Differentiation Trees of Embryos

**DOI:** 10.3390/biology5030033

**Published:** 2016-08-18

**Authors:** Bradly Alicea, Richard Gordon

**Affiliations:** 1 Orthogonal Research, 1408 Rosewood Drive, Champaign, IL 61821, USA; 2 OpenWorm Foundation, Cyberspace, San Diego, CA 92110, USA; 3 Gulf Specimen Marine Laboratory & Aquarium, 222 Clark Drive, Panacea, FL 32346, USA; DickGordonCan@gmail.com; 4 C.S. Mott Center for Human Growth & Development, Department of Obstetrics & Gynecology, Wayne State University, 275 E. Hancock, Detroit, MI 48201, USA

**Keywords:** developmental biology, computational biology, lineage trees, embryogenesis, biological complexity

## Abstract

Embryonic development proceeds through a series of differentiation events. The mosaic version of this process (binary cell divisions) can be analyzed by comparing early development of *Ciona* *intestinalis* and *Caenorhabditis elegans*. To do this, we reorganize lineage trees into differentiation trees using the graph theory ordering of relative cell volume. Lineage and differentiation trees provide us with means to classify each cell using binary codes. Extracting data characterizing lineage tree position, cell volume, and nucleus position for each cell during early embryogenesis, we conduct several statistical analyses, both within and between taxa. We compare both cell volume distributions and cell volume across developmental time within and between single species and assess differences between lineage tree and differentiation tree orderings. This enhances our understanding of the differentiation events in a model of pure mosaic embryogenesis and its relationship to evolutionary conservation. We also contribute several new techniques for assessing both differences between lineage trees and differentiation trees, and differences between differentiation trees of different species. The results suggest that at the level of differentiation trees, there are broad similarities between distantly related mosaic embryos that might be essential to understanding evolutionary change and phylogeny reconstruction. Differentiation trees may therefore provide a basis for an Evo-Devo Postmodern Synthesis.

## 1. Introduction

A central issue in the evolution of development involves how a diversity of phenotypes arose from a presumably universal set of mechanisms. In regulative development, a cell becomes a specific type because of its position at a given time in development, i.e. transplantations of patches of tissue that are early enough can alter the fate of its cells. In mosaic development, each cell has a specific fate. This is seen most dramatically if an early embryo is cut in two: regulative embryos produce two smaller, but whole embryos [1], whereas mosaic embryos produce two half embryos [2]. Most organisms exhibit both kinds of development at some stage [3,4,5,6,7]. These modes of development are not mutually exclusive, as a given taxon can often exhibit both modes at distinct stages of embryogenesis. There is great diversity across species: amphibian embryos are highly regulative at early stages, while early *Amphioxus* and tunicate embryos are highly mosaic. Yet both produce similar tissues and have similar fate maps [8]. Organisms that develop in a mosaic fashion also tend to be eutelic [7,9], having a fixed number of cells per adult individual across a particular species, whereas regulating embryos easily adjust to the number of cells available, whether varied by cutting [1] or by ploidy [10].

We can characterize the process of differentiation by means of a lineage tree, where each descendent cell produces two descendent cells and so on until formation of the organism is complete. In mosaic organisms, most or all of these cell divisions are asymmetric, producing two different cells that also differ from their mother cell. In regulating embryos, cell division and cell differentiation appear to be uncoupled, allowing for multiple cells of the same type. Exceptions to the usefulness of the lineage tree in representing development occurs in a number of specific cases. One of these cases involves the fusion of cells, as is the case during skeletal muscle formation [11]. This particular situation arises in later development, or in syncytial stages at the beginning of embryogenesis, as in *Drosophila* [12]. For strictly mosaic organisms, the lineage tree is the same for all individuals. In this case, each cell can be given a unique name.

As an alternative to the lineage tree, we can use additional information from the developing embryo to construct a differentiation tree [10,13,14]. For strictly mosaic organisms, the differentiation tree is just a rearrangement of the branches of the lineage tree, changing their order left to right across the page. We will specify both orderings shortly. To hone our thoughts, we will use the language of graph theory [15]. A directed acyclic graph (DAG) [16,17], is a set of points that form a graph which contains no cycle. Lineage trees and differentiation trees can both be considered DAGs. When they are laid out on a plane so that their edges do not cross, both types of trees are also planar graphs [18] and could be called “planar trees”. If a tree starts at one point, called its “root”, as ours do with the fertilized egg, it is called a “rooted tree” [15,19,20]. The plane onto which we map our rooted trees has two coordinates. An asymmetric cell division can be represented as a bifurcation, so that lineage trees and differentiation trees are “binary trees” [21].

As with lineage trees, developmental time is represented along the vertical axis of a differentiation tree. This coordinate need not be linear with real time, as it may reflect developmental stages, whose timing is temperature dependent for poikilotherms. However, because time does not go backwards, it is an example of an “upward drawn” tree [22], for which a number of aesthetically pleasing criteria, including “generating congruent drawings for isomorphic subtrees”, can be designed and met with practical algorithms [23]. However, new algorithms may be needed for optimal layout of our trees (cf. [24]), because the placement of nodes in the vertical time axis cannot be at discrete “horizontal lines according to their level (graph theoretic distance from the root)” [23]. (cf. [25]). It is possible for each node to have its own level, i.e., time of occurrence. We use the word “depth” to describe the number of tree edges from the root to a given node, since in continuous time along the vertical axis “level” and “depth” are not synonymous. In other words, *depth* is an integer variable, and *level* is a continuous real variable.

The horizontal component of both lineage and differentiation trees derived from mosaic embryos rely on the concept of an “ordered binary tree”. Lineage trees are generally ordered with respect to the anatomy of the developing embryo. For instance, at a bifurcation, the cell closer to the anterior end is placed on the left, and the cell of the pair closest to the posterior end is placed to the right. In the case of a differentiation tree, the smaller cell resulting from a division of unequal size branches to the left and the larger cell branches to the right. Throughout the manuscript, we will refer to this as an asymmetric division, however, this definition of asymmetric is solely based on size after cell division, and is distinct from the functional criteria discussed in [26].

**Figure 1:**
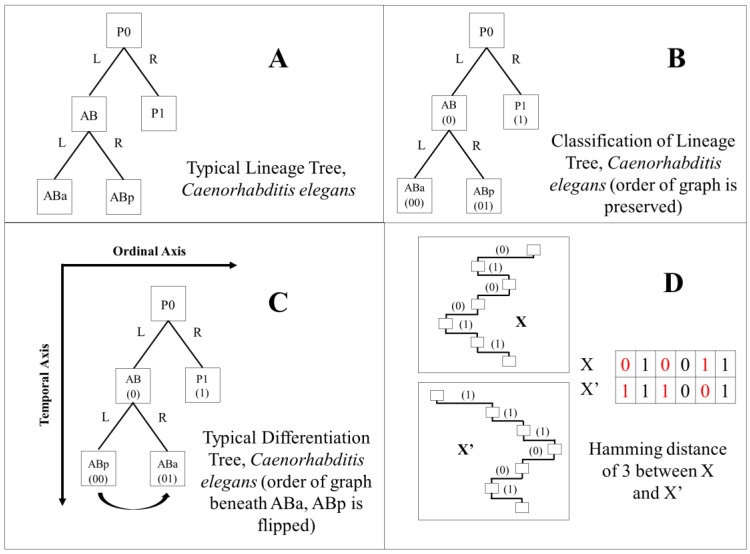
A demonstration of how binary codes produce different classifications of each cell in *C. elegans* embryos according to two different binary tree orderings. (**A**) Five nodes and their left/right (L/R) ordering in the *C. elegans* lineage tree; (**B**) a binary lineage code classification for these same nodes (cells) in the lineage tree, with 0 representing nodes that branch to the left, and 1 representing nodes that branch to the right; (**C**) the same five nodes as shown in A and B, but reordered to reflect their relative cell volumes, small daughter cells to the left (0) and large daughter cells to the right (1). This is reflected in their differentiation code classification; (**D**) a demonstration of how the Hamming distance metric is calculated for the “distance” between the lineage code and the differentiation code for a given cell (X and X’). Only the path in the tree is shown, from the root (top) to the given cell (bottom). Represented as a decimal number, the Hamming distance (see Methods) is the number of differences (or flipped bits) between X and X’ (labeled in red).

In summary, lineage and differentiation trees differ from conventional graphs in that one axis represents the timing of developmental events. Both the lineage tree and the differentiation tree for the same mosaic organism are both rooted, planar, ordered binary trees, but differing only along their ordinal axis (perpendicular to the time axis). See Figure 1C for a visual example. They are therefore isomorphic in the graph theoretical sense [27]. Two isomorphic differentiation trees that look the same except for changes in their timing along the vertical axis have been hypothesized to describe heterochrony [10,13]. When two trees are not isomorphic, we shall say that they differ in “topology”. One example of this is that lineage trees of mutants often differ in topology [10,28,29,30].

Lineage trees and differentiation trees provide us with a means of looking at general trends of embryogenesis. Not only is there potential to study phenomena such as developmental module formation [31,32] at multiple scales, but also organizational principles such as early left-right asymmetry [32] and whether or not universal processes occur across the regulative/mosaic spectrum, such as we have hypothesized [10,13,14]. Furthermore, differentiation trees may be duplicated in a manner similar to genome duplication [12]. This, along with the comparative power of the differentiation tree and associated differentiation code, can reveal important characteristics regarding a species’ evolutionary history. The Modern Synthesis was based on allele changes in populations, and eschewed both embryology and gene duplication (§10.06 in [13,33]. There have been recent calls for a Postmodern Synthesis taking these and other phenomena into account [33,34]. In our previous work we proposed that: “phylogeny reconstruction or taxonomy should be based on the differentiation trees of organisms” [13]. The Tree of Life, at least for multicellular organisms, would then become a tree of differentiation trees. By viewing hierarchical patterns of differentiation themselves as conserved evolutionary phenomena, our work serves to complement current molecular phylogenetic approaches. In previous work, we suggested that the differentiation tree is a universal feature of embryogenesis [10,13]. Here we begin to test this idea.

Our reference points will be two mosaic species from rather disparate taxa: the chordate sea squirt *Ciona intestinalis*, and the nematode roundworm *Caenorhabditis elegans* (henceforth referred to as *Ciona* and *C. elegans*, respectively). This comparative analysis is restricted to the mosaic parts of each lineage tree: up to 10 division events in *C. elegans* (pre-hatch embryo, excluding germ and post-embryonic cells), and the pre-gastrulation embryo in *Ciona* (112-cell stage). We use secondary biometric data that include cell volume, cell nucleus location, and lineage tree identification to allow us to make comparisons between them at early stages of embryonic development. We then look at the commonalities between these two taxa to gain a greater appreciation for what may be essential features of mosaic development. Here we make a start on comparing these two mosaic species with the differentiation tree [10,35] of a regulating embryo, the urodele amphibian *Ambystoma mexicanum* (also known as the axolotl).

While *C. elegans* has many advantages as a model of mosaic development [36], species in the genus *Ciona* give us insights into chordate embryogenesis [37]. More generally, ascidian model organisms can provide information about the evolutionary differences between various instances of mosaic development. In a cellular-level comparison of the tunicates *Phallusia mammillata* and *Oikopleura dioica* embryogenesis, evolutionary conservation dominates the mosaic process. However, there are also instances of evolutionary innovations related to the timing of differentiation events and alterations in morphogenetic movements [38]. Since mosaic development appears in so many developmental contexts, it is likely to have evolved multiple times in different evolutionary contexts (e.g., parallel evolution) [38,39].

In this paper, we try to answer three questions regarding the evolution of development. (1) What parameters we can extract that would contribute to building a minimal mosaic phenotype? This might allow us to begin hypothesizing about the phenotypic properties of a mosaic development common ancestor; (2) Are there common features of mosaic development? As our examples are from different Orders in the tree of life, they do not represent shared evolutionary histories [40]. Nevertheless, perhaps there is a series of “must-haves” for any potential evolutionary path to mosaic development; (3) At what level of organization are lineage or differentiation trees comparable across the animal Tree of Life? The similarity of fate maps across phyla [8] encourages us to adopt innovative methods in the service of understanding these issues.

### Higher-Order Patterns and Organization by Differentiation

In making a cross-species comparison, we anticipate a number of higher-order patterns in the mosaic developmental program that are informative of both spatial organization in the emerging phenotype and the formation of higher-order anatomical structures. For example, comparisons of muscle fate across ascidian genera (*Ciona intestinalis* and *Halocynthia roretzi*) demonstrate variability despite both species having highly-invariant mosaic development with respect to individuals [41]. We should also expect to uncover many regularities within single species that either may or may not be found in other species. Bao et al. [42] and Ho et al. [43] report that cell division timing is highly regular among individual embryos of the same species. These authors also suggest that the rate of cell division may vary according to a cell’s fate [42]. Another potential regularity is the existence of power-law scaling between the division times of cells and their size. It has been found that the process of embryogenetic cell differentiation in *C. elegans* is both scale-invariant [44] and conforms to a power law [45]. Such regularities may be due to a general mechanism common to all species with a certain mode of development, or might result from species-specific developmental histories [46].

One aspect of embryogenesis that provides clues to the patterns that underlie both interspecific variation and intraspecific invariance is the volumes of daughter cells as they recursively divide from a one-cell precursor, the fertilized egg or zygote. While we expect general trends in cell volume over the course of development, asymmetries in volume between daughter cells originating from the same mother cell confound potentially global trends, especially as compounded from one generation of cells to the next. The published *C. elegans* lineage tree places daughter cells left or right according to their position along the embryo’s posterior/anterior axis [10,47]. Based on the presence of volume differences between sibling cells in a binary division, we can reorganize the lineage tree into a differentiation tree. A differentiation tree for a mosaic organism is organized on the basis of smaller daughter cells being represented by leftward branches and larger daughter cells represented by rightward branches [10,13,14]. This rearrangement of the lineage tree by an alternative criterion than cell position provides comparative information between sub-lineages, and perhaps clues as to the role of cell volume asymmetry in causing and propagating regional differentiation processes in the embryo.

In mosaic organisms, cellular differentiation proceeds through a branching sequence of binary divisions which we have illustrated using both the *Ciona* (Figure S1A, tissue types distinguished by color; Figure S1B, no tissue type distinction made) and *C. elegans* (Figure S2A, tissue types distinguished by color; Figure S2B, no tissue type distinction made) datasets. While the nomenclature of the lineage tree is based on these division events, we can also use a binary “lineage code” to classify the asymmetry of these division events. This can be done for both the existing *C. elegans* and *Ciona* lineage trees. Similarly we can define a differentiation tree and a corresponding “differentiation code” for each species (for an example, see Figure 1, Panels A–D). We assume that differentiation trees informative of the mosaic embryogenetic process can be constructed by ordering each daughter cell pair by relative size, although a correlation between cells being of different types and sizes may not always exist [48]. This requires identifying the smaller cell and the larger cell of the pair, which is analogous to the tissues generated by contraction waves and expansion waves, respectively, that result from the differentiation process in regulative embryos [35]. This allows us to focus on this aspect of variation as a driver of temporal and spatial patterns within the early embryo. Yet by comparing across taxa and using a relatively limited indicator of variation, we can also discover some invariant properties of mosaic differentiation.

Using the differentiation code to construct a differentiation tree, particularly in comparison to a lineage tree, may ultimately allow us to find evidence regarding applicability of the phenomenon of differentiation waves to mosaic organisms [13]. Differentiation waves are hypothesized to serve as the signal that causes individual cells to differentiate in a coordinated spatiotemporal manner. They have been observed as early as the Stage 8 blastula [49] in the chordate axolotl (*Ambystoma mexicanum*) regulating embryo [35,50,51]. There are two general types of differentiation waves: contraction and expansion, involving contraction or expansion of the cell state splitter, a cytoskeletal apparatus in epithelia that both triggers a step of differentiation and propagates as a wave from cell to neighboring cell [14] (there are six variants on these waves [10]). The bridge to mosaic organisms is made in theory by assuming that the trajectory of a differentiation wave involves many cells in regulating embryos, but only one cell in mosaic organisms [13]. Thus when a cell in a mosaic organism divides asymmetrically, the larger daughter cell is presumed to have gone through a one-cell expansion wave, while the smaller daughter cell is presumed to have gone through a one-cell contraction wave. The differentiation tree and its associated differentiation code provide a means to compare mosaic and regulative development.

## 2. Materials and Methods

### 2.1. Description of Datasets, Ciona Intestinalis

**Figure 2:**
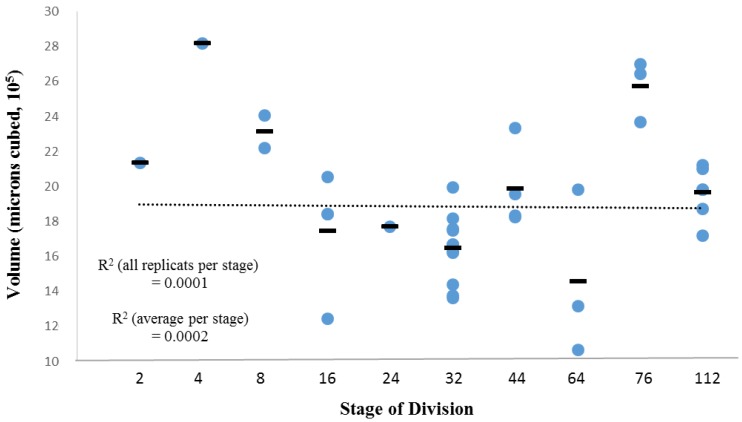
Embryo volume per cell stage of embryogenesis for *Ciona*. Volume (measured in cubic microns) is averaged over a number of observations (N). N (stage) is given in Table S1.

This representative ascidian (tunicate, Urochordata) is an entirely hermaphroditic species that has 1579 cells at the mid-tailbud stage [52]. Datasets [8,53] consist of pre-gastrulation cell divisions (up to 112-cell stage). Biometric data for each cell includes cell names (identity) and percentage of total embryo volume for the 2-, 4-, 8-, 16-, 24-, 32-, 44-, 64-, 76-, and 112-cell stages (data available from [54]). Volume for each cell is calculated as a percentage of the entire embryo at that stage. Figure 2 demonstrates the total volume for all embryos (per stage) in the available dataset, which is calculated as the sum of the cell volumes of all the cells at a given stage. While there is technical variation in the data for total embryo size, there is no strong trend, and so we can calculate the average volume, independent of stage, over this range of stages, as 2.04 × 10^6^ µm^3^. In the original acquisition of the data, a tracer enzyme was used to identify each cell body in a whole embryo. All data (cell identities and volumetric measurements) from each embryonic stage were integrated into a single data structure in order to determine line of descent based on a reference lineage tree [8], confidence intervals, and differentiation tree order.

### 2.2. Description of Datasets, Caenorhabditis elegans

This representative nematode (roundworm, Nematoda) is a usually hermaphroditic organism that has 959 cells in the adult hermaphrodite and 1031 in the adult male. Datasets consist of pre-hatching cell divisions (up to 558 cells) [55]. In the original acquisition of the data, identification of the cell nucleus was accomplished using a GFP^+^ marker. Three-dimensional (spatial) position, diameter, and nomenclature (identity) for all cells are extracted from observations of 261 embryos. These variables are then averaged over every observation of a specific cell type. Discrete GFP^+^ regions are segmented from microscopy images using computer vision techniques, and are used to construct an optimal spherical representation (see [55]). Due to the nature of the quantification of the *C. elegans* data, corresponding volumetric data of the whole embryo (as we have for *Ciona* in Figure 2) is not available. This spherical representation is based on identification of the centroid and annulus for these segmented regions from florescence signal intensity. While this is not an exact measure of diameter for the entire cell, it provides a reasonable approximation. The positional data is translated to have its origin at the location of the P0 cell, and is used to produce a three-dimensional spatial representation of the embryo. In this representation, orientation along A-P axis is reversed so that negative values are closer to the anterior end.

### 2.3. Methods for Collecting Secondary Data

Embryos for *Ciona* are incubated at 18–20 °C [56], while the cell body is imaged using an antibody stain. *C. elegans* embryos are incubated at 25 °C [57], while the cell nucleus is imaged using a GFP^+^ marker. These techniques allow for approximation of volume by calculation of cell volume as a proportion of total embryo volume in *Ciona* and cell volume as a calculation of a sphere based on an approximation of cell diameter for *C. elegans*.

### 2.4. Finding Biologically-Meaningful Asymmetric Divisions

A model-free confidence interval [58] is used to assess the existence of a biologically-meaningful size asymmetry resulting from single cell division events. We determine whether or not a daughter cell pair is above a defined confidence interval threshold by using an absolute threshold relative to the volume differential between two daughter cells. The confidence interval is defined as:

(1) $$G=(\frac{\left|C_{i}-\;C_{j}\right|}{2\;\left(C_{i}+\;C_{j}\right)})\cdot h$$

where *G* is the difference criterion, $C_{L}$ is the larger daughter cell volume in the unordered pair *C_i, j,_* *C_s_* is the smaller daughter cell volume in the unordered pair *C_i, j,_* and *h* is the threshold. A range of *t* values (0.05, 0.25, 0.50) are calculated for cells as a percentage of volume in the *Ciona* embryo (Table S2). A range of *t* values (0.01, 0.025, 0.05) are calculated for a spherical approximation of cell volume in the *C. elegans* embryo (Table S3). This difference criterion (*G*) is compared to the difference in volume between *C_i_* and *C_j_* in the following manner:

if *G* > *h*, then *g* = 1 else *g* = 0 (2)

where *g* denotes the success (1) or failure (0) of the statistical test. If a difference exceeds the bounds of the confidence interval for a specific daughter cell pair, then it is considered to be a robust indicator of biological difference. For an empirical demonstration, see Tables S2 (*Ciona*) and S3 (*C. elegans*).

### 2.5. Ratio Between Larger and Smaller Daughter Cells

The size ratio of a daughter cell pair is calculated by dividing the volume of the smaller cell (numerator) by the volume of the larger cell (denominator). This provides us with an index where a symmetrical daughter cell pair is equal to 1, and larger volume asymmetries result in decreasing values asymptotic to 0.

### 2.6. Differentiation Trees

Lineage and differentiation trees [13] are assembled and annotated in PowerPoint and GIMP [59]. We have published differentiation trees for *Ciona* [60] and *C. elegans* [61]. Static versions can be found in Figure S1A and S1B (*Ciona*) and Figure S2A and S2B (*C. elegans*). The differentiation tree of the *C. elegans* embryo includes cell lineages up to 10 division events, with data from lineage trees and fate maps of Sulston et al. [26], Hobert [62,63], and Goldstein et al. [64]. Division event timing was approximated from Bhatla [65]. Cell diameter information was approximated from Bao et al. [55] and according to the methods described above. The differentiation tree of the *Ciona* embryo includes cell lineages from the one-cell to the 112-cell stage (10 division events), with data from lineage trees and fate maps from Nishida [66], Nishida and Stach [8], and Tassy et al. [53]. Data for *Ciona* cell volume approximations is from the ANISEED database [53].

For both differentiation trees, a division event occurs when a mother cell divides into two daughter cells. In some cases one of the daughter cells is terminal, and either ceases further differentiation or disintegrates by apoptosis. According to the differentiation tree convention, smaller cells are placed to the left and larger cells are placed to the right. Each cell (except for the one-cell stage in both *Ciona* and *C. elegans* and the two and four-cell stage cells in *C. elegans*) is annotated with its standard nomenclature and a decimal value that describes the relative volume of each daughter cell (e.g., smaller cell is 0.49, larger cell is 0.51 volume relative to whole embryo).

### 2.7. Differentiation Code and Tree Classification

Differentiation trees were constructed by using the binary differentiation events as described by Sulston et al. (*C. elegans*) [26] and Nishida (*Ciona*) [66]. To arrive at a differentiation code, each daughter cell in a pair is classified as either “0” or “1”, dependent upon each cell’s relative volume. This classification is done independently of Equations (1) and (2). The differentiation code is a sequence of binary digits that provide an absolute address in terms of lateral position (left-right) and depth (number of branches from the root) in the differentiation tree ordering. Lineage trees are coded according to their published arrangement in the lineage tree. Cells organized to the left are classified using a “0”, while cells organized to the right are classified using a “1”. The address system is the same as that of the differentiation tree, with the exception that the “lineage code” is in a different order with respect to the differentiation tree ordering. See Tables S4 (*Ciona*) and S5 (*C. elegans*) for more information.

### 2.8. Composite Differentiation Code

In order to address inconsistencies in size differences at the two and four-cell stage of our *C. elegans* secondary dataset [51], we generated a composite differentiation code. This combined knowledge of relative cell size at the two and four-cell stage from cell polarity studies [64,67] with our dataset of cell volumes estimated from GFP^+^ nuclei. This composite differentiation code was used to estimate the *C. elegans* differentiation tree topology (Figures S2A and S2B), and can be found in Table S5.

### 2.9. Power Regression Formula

The power regression is a form of nonlinear curve-fitting used to detect biological power laws [68] and other nonlinear phenomena. The power function can be defined as

(3) $$y=\;mx^{b}$$

where log(*m*) is the intercept and *b* is the slope on a semilog plot of *y* versus *x*.

### 2.10. Hamming Distance Calculation

The Hamming distance metric [69] is used to calculate a distance between differentiation or lineage codes for two cells with the same depth (see Figure 1, panel D). The Hamming distance can be defined in the following manner:

(4) $$D_{H}=\overset{k}{\underset{i=\;1}{\sum }}\left|x_{i}-\;y_{i}\right|$$

where *x_i_* and *y_i_* are binary strings of the same length (depth in the tree) compared pairwise, *k* is the length of both strings, and DH counts the number of differences between the two strings in terms of bits. This calculation can be done for the same cell, in regards to its positions in the lineage and differentiation trees, between different cells in the same tree, that are at the same depth, or between cells at the same depth in trees of two different organisms.

In this paper, we calculate the Hamming distance between the orderings provided by the lineage tree representation versus the differentiation tree representation. This is done to provide a quantitative measure of how ordering the line of cellular descent by different criteria changes the relationships between these trees. Each comparison is made on a unique cell as defined by the Sulston et al. nomenclature [26]. A unique cell will always have the same depth, but a different binary code of the same length, which is dependent upon the tree ordering. See Tables S4 (*Ciona*) and S5 (*C. elegans*) for more information.

### 2.11. Three-Dimensional Representation of Hamming Distances in C. elegans

A three-dimensional scatter plot (Figure S4) is used to visualize the relationship between Hamming distances between the lineage and differentiation tree for each cell in the 128-terminal cell embryo (N = 230), and a cell’s spatial location in the *C. elegans* embryo. This corresponds to nine (9) division events for the differentiation tree in Figures S2A and S2B. The H abbreviation in Supplemental Figure 4 (*C. elegans*) and Tables S4 (*Ciona*) and S5 (*C. elegans*) stands for Hamming distance. All cells in Figure S4 have a lineage depth that ranges from one to seven division events away from the one-cell stage (P0).

### 2.12. CAST (Cell Alignment Search Tool) Analysis

The binary codes for any two cells can be compared in a manner analogous to BLASTing, where BLAST is the Basic Local Alignment Search Tool [70]. We call this CASTing, where CAST = Cell Alignment Search Tool. The binary code of a cell can be written as D.b1, … bD. The prefix (D) is a decimal number representing the lineage depth of a cell. The suffix is its binary code. Here we only apply CASTing to differentiation codes. For the differentiation code, the binary digit *b_i_* = 0 for the small cell in an asymmetric division or for a contraction wave in a regulating embryo [10,13,14], or *b_i_* = 1 for the larger cell or an expansion wave. When it is easier to think in terms of letters rather than binary numbers, one may substitute C = 0 and E = 1. BLASTing works on the nucleotide or amino acid sequence, represented by letters, so the CAST analogy to BLAST is clearer when the differentiation code is expressed in letters.

A CAST analysis is done by aligning two sets of CAST sequences in a manner based on the Needleman-Wunsch method of genome alignment [71]. The CAST score is a recursive summation calculated by adding a point for a match between the two differentiation code sequences, and subtracting a point for a gap, or a point in the alignment where only one of the two differentiation code sequences contains information. Unmatched CAST codes will be unique to their differentiation tree, and are to be expected in cross-species comparisons as not all lineage trees have the same topology. A demonstration of the alignment method and the scoring is provided in Table S6.

### 2.13. Graphs and Analysis

All graphs were constructed and statistical analyses conducted in MATLAB and Excel. All publically-available data are available in the following Supplemental Tables (Excel format): Table S2 (*Ciona* Confidence Interval Data), Table S3 (*C. elegans* Confidence Interval data), Table S4 (*Ciona* Differentiation and Lineage Codes), Table S5 (*C. elegans* Differentiation and Lineage Codes), Table S6 (CAST Codes for *Ciona*, *C. elegans*, and Axolotl).

## 3. Results

### 3.1. Introduction to Analysis

Our analysis will feature a variety of analyses that correspond to our three questions posed in the Introduction: within-species cellular variation, cellular variation across lineages, and a comparison of lineage and differentiation trees. (1) To examine within-species cellular variation, we produce two analytical results. The first is to establish the developmental context by looking at the relationships between cell volume and temporal events in development. Then, we present a means to distinguish variation exhibited in the data from biological stochasticity; (2) We investigate cellular variation more generally across lineages by conducting further analyses on cell volume information. This is done using two criteria: the lineage depth of a given cell, and the lineage depth of all smaller cells resulting from a single binary division versus all larger cells resulting from single binary divisions; (3) Finally, we construct and analyze differentiation trees as a contrast to lineage trees, computing both differentiation codes and lineage codes. A metric from information-theory (Hamming distance) is used to assess the importance of cell position at each depth of the two tree structures. Considering differentiation codes as their own source of information, we generate and compare CAST (Cell Alignment Search Tool) codes across three species.

### 3.2. Within-Species Embryonic Variation

The first set of analyses we conducted between our *Ciona* and *C. elegans* species involves understanding the relative variation within each species. The analysis is a means to find commonalities between species, while also validating the data through consistency with other studies. We accomplish this in two ways. One way is to investigate the relationship between the volume of a cell with the lifetime (length of cell cycle) of that same cell. The lifetime of a cell is the length of time between its origin from a mother cell and its division into two daughter cells. A second way is to estimate the volumes of daughter cells after division, and assign a quantitative criterion to the relative difference in volume between the two. This confidence interval (see Methods) helps to eliminate false positive results that arise from technical variation in the cell volume approximations.

**Figure 3:**
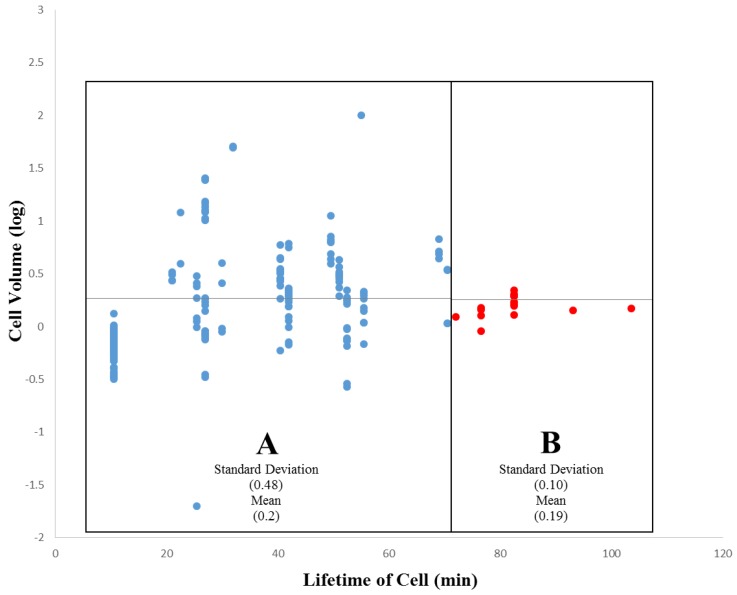
The log-linear relationship between cell volume (log_10_ of cell volume in µm^3^) and the cell lifetime (linear) of individual cells for *Ciona* embryo (raised at 18–20 °C) based on all cells between the one-cell stage and the 112-cell stage (N = 225). Cell volume is normalized as a percentage of total embryo volume. Some data points are overlapping, and are divided into two categories. (**A**) Blue dots (n = 209), x-axis interval 10.5–70.5 min; (**B**) red dots (n = 16), x-axis intervals 72–103.5 min. The mean value for category A is 0.20, and the mean value for category B is 0.19, shown on the graph as two horizontal gray lines.

**Figure 4:**
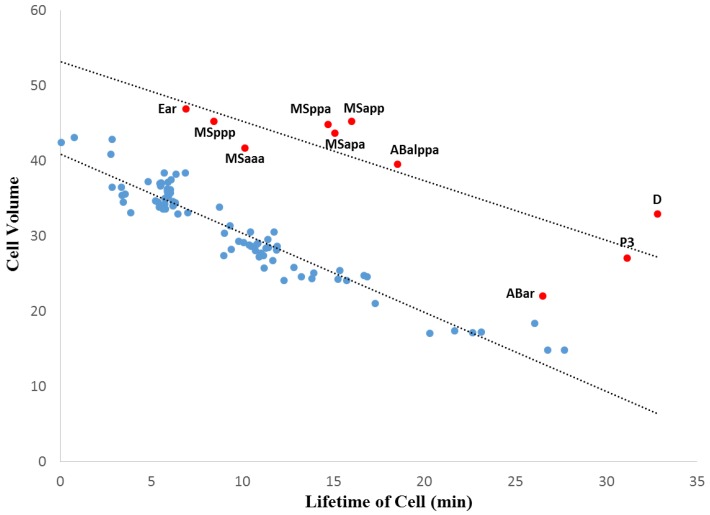
The relationship between cell volume and the cell lifetime of individual cells for the pre-hatch *C. elegans* embryo (raised at 25 °C), based on 96 cells (N = 96). Cell volume (µm^3^) extrapolated from cell diameter after normalization to P0 (1-cell stage). Main data series is blue, selected outliers are red. The data from the outliers is not included in the lower least squares fit, and vice versa.

Initially, we will examine the relationship between cell volume and the cell lifetime of that cell, and test the hypothesis that smaller cells have longer cell lifetimes than larger cells. The relative cell volume versus cell lifetime data for Figure 3 was extracted from the core secondary data for *Ciona* (see Methods), while Figure 4 was assembled from the core secondary data for *C. elegans* (see Methods) and an additional dataset provided in [42]. These data sets represent the first 180–300 min into *Ciona* embryogenesis, and the first 200 min of embryogenesis in *C. elegans*, respectively.

Figure 3 (*Ciona*) demonstrates a bimodal relationship between cell volume and the lifetime of a cell (from division to division), while Figure 4 (*C. elegans*) demonstrates a negative linear relationship between cell volume and the lifetime of a cell. In both *Ciona* and *C. elegans*, smaller cells tend to have longer lifetimes, while larger cells tend to have shorter lifetimes. This is consistent with the manner in which differentiation proceeds in *Ciona*: larger cells divide into two cells, each roughly half the volume of the mother. We also stratified the data by division event (level in the lineage tree) to rule out the effects of developmental stage. However, plotting the data in this way does not reveal any additional information regarding the cell volume/lifetime of cell relationship neither for *Ciona* (Figure 3) nor *C. elegans* (Figure 4). The structure of this relationship differs by species. In *Ciona* (Figure 3), the cell volume/cell lifetime relationship exhibits two distinct phases: in the first phase (A), cells exhibit significant variation in cell volume with respect to cell lifetime (standard deviation = 0.48 µm^3^), while the other phase (B), cell volume exhibits much less variation (standard deviation = 0.10 µm^3^). While the boundary between phases is somewhat arbitrary, we can say that longer lived cells (with a lifetime of roughly 70 min or older) exhibit less variability in cell volume. Although we cannot establish a direct link to tissue formation, given currently available data, it provides at least some evidence for cellular sublineages that behave somewhat differently from the rest of the embryo.

In *C. elegans* (Figure 4), we observe quite a different pattern for the relationship between cell volume and the lifetime of a cell. Figure 4 demonstrates a negative linear relationship (blue) with selected outliers (red) that also produce a negative linear trend. The main data series (blue) yields a strong linear regression (R^2^ = 0.91, *p* > 0.001). In general, the longer the lifetime of a cell, the smaller its volume. In identifying 11 selected outlier cells (points greater than 2.5 standard deviations away from the main data series trend line), we can see that eight (8) of these cells are descendants of P1 (clustered in the MS lineage). Only a single outlier in Figure 4 is descended from AB (ABar). Overall, however, the variation observed in *C. elegans* is much less than in *Ciona*.

In both *Ciona* and *C. elegans*, smaller cells tend to have longer lifetimes, while larger cells tend to have shorter lifetimes. This is consistent with the manner in which differentiation proceeds: larger cells differentiate into two smaller cells, each roughly half the volume of the mother. However, the structure of this relationship differs by species. These data are suggestive of a number of possibilities. Perhaps a fundamental limit exists that prevents larger cells from having exceedingly long lifetimes. The distribution of both division times and cell lifetimes is consistent with the overall unfolding of embryonic development: larger cells tend to occur closer to the root of the tree (e.g., 1-cell stage). A plot for division time versus cell lifetime can be found in Figure S3 for both *Ciona* (top) and *C. elegans* (bottom). These plots reveal species-specific patterns of variation. In the case of *Ciona*, cell lifetime converges to a single value for the two-cell, four-cell, and 112-cell stage. All other stages are much more variable with respect to cell lifetime, with maximal variation in the 44-cell stage. For *C. elegans*, we observe a positive logarithmic tendency with a fairly large amount of variation for the cell lifetime data after the two-cell stage (AB, P1).

While Figure 3 and Figure 4 indeed confirm that smaller cells have longer cell lifetimes than larger cells, we would also like to clarify the role of constraints imposed upon a cell by its genealogical context. To do this, we must present these data in the developmental context. We do this by approximating the linear relationship between cell volume and time of division in embryonic development.

**Figure 5:**
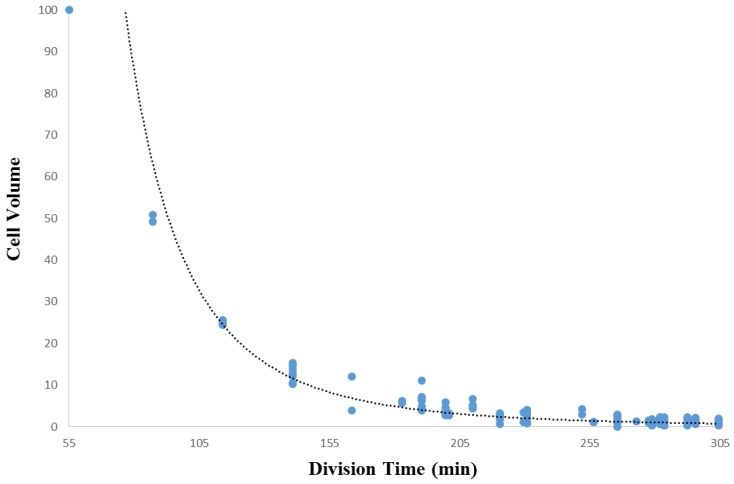
An analysis of cell volume versus lifetime in *Ciona*, based on all cells between the one-cell stage and the 112-cell stage (N = 224). Cell volume is normalized as a percentage of total embryo volume, and lifetime is the duration of the period of time between a cell’s creation via division and its division into two daughter cells. Trendline (black) is based on a power function.

**Figure 6:**
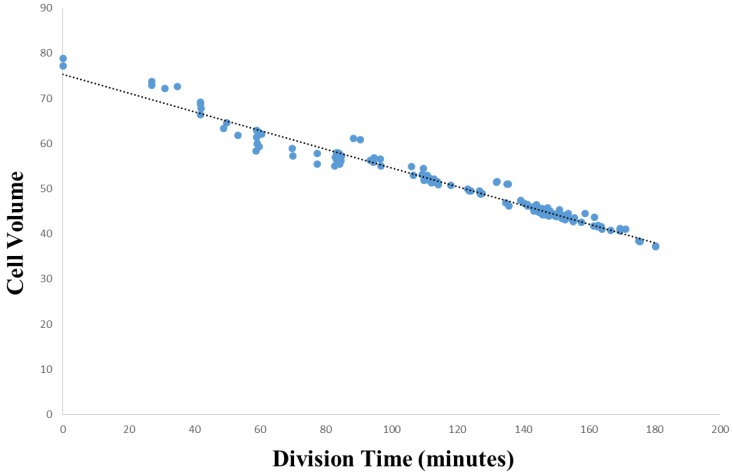
An analysis of cell volume in *C. elegans* versus lifetime (as calculated by Bao et al. [55]), based on 192 cells (N = 192) from a subtree of the AB lineage. Cell volume is extrapolated from nuclear diameter and is normalized as a percentage of total embryo volume Lifetime as in Figure 5. Trendline (black) is based on a linear function.

An analysis of cell volume in the context of development is shown in Figure 5 (for *Ciona*) and Figure 6 (for *C. elegans*). The baseline division time (0 min) is calculated relative to the time of fertilization in *Ciona*, and the beginning of the two-cell stage in *C. elegans*. The source of data for Figure 5 and Figure 6 are identical to those of Figure 3 and Figure 4, respectively. Figure 6 is also based on timing information published by Bao et al. [42]. Using a regression analysis, the comparison between cell volume and division timing yields nonlinear and linear relationships for *Ciona* and *C. elegans*, respectively. Figure 5 reveals a moderately strong power function (see Methods, Section 2.8) for *Ciona* (*R*^2^ = 0.70, *p* < 0.001), while Figure 6 yields a much stronger relationship for *C. elegans* (*R*^2^ = 0.97, *p* < 0.001). While there are more outliers in the *Ciona* example, this could be due to a number of technical factors such as more precise temporal measurements for the *C. elegans* example and differences in how the volume measurement is approximated between these species.

Next, we would like to more explicitly understand the role of variation in volume as a consequence of developmental processes. This can be done by characterizing asymmetric cell divisions as a statistical distinction between slight variations in size asymmetry between daughter cells and variations more likely to be biologically significant. We accomplish this by defining a confidence interval threshold for the data, and then comparing four different thresholds in an attempt to understand how many asymmetrical cell divisions are biologically meaningful and not due to natural (or technical) variation. Low levels of difference (e.g., 0.05 in *Ciona*) should provide a large proportion of above threshold events. However, increasing the threshold should result in a smaller proportion of above-threshold events.

Table 1 shows the results for four distinct confidence interval thresholds in *Ciona*, while Table 2 shows the results for four distinct confidence interval thresholds in *C. elegans*. One notable result is that the confidence interval thresholds in Table 1 and Table 2 exhibit an order of magnitude difference, even though the pattern of their performances on the data remain similar. Whether this is explicable by the relative lack of cell volume variation in *C. elegans* relative to *Ciona* is unclear. The explanation cannot simply be due to a difference in approximating cell volume, however, because comparisons between daughter cells are local observations involving two cells.

As expected, lower confidence interval thresholds (e.g., 0.005 as compared to 0.50) result in a larger number of daughter pairs above the confidence interval threshold. Note that this analysis does not speak to whether a given cell division is asymmetric in terms of generating two different kinds of daughter cells—it only tells us the confidence with which we can determine if the daughter cells differ in volume. Since the ordering in a differentiation tree depends on volume differences, this places limitations on the accuracy with which we can derive the differentiation tree from such data.

### 3.3. Cellular Variation Across Lineage Trees

We next examine trends in cell volume in the context of the lineage tree for both *Ciona* and *C. elegans*. We can extract this trend in at least two ways: a calculation of a given cell’s lineage depth, and a calculation of the ratio of smaller daughter cell size to larger daughter cell size across all binary divisions. A cell’s lineage depth refers to its genealogical position from the one-cell stage. Using the *C. elegans* lineage tree as an example, cell ABal would have a lineage depth of three (3), and corresponds to two division events from cell AB. In terms of comparing smaller and larger cells in each division, we can evaluate each division event and its corresponding daughter cells. The ratio between their relative sizes can be characterized by lineage depth for both *Ciona* and *C. elegans*.

**Figure 7:**
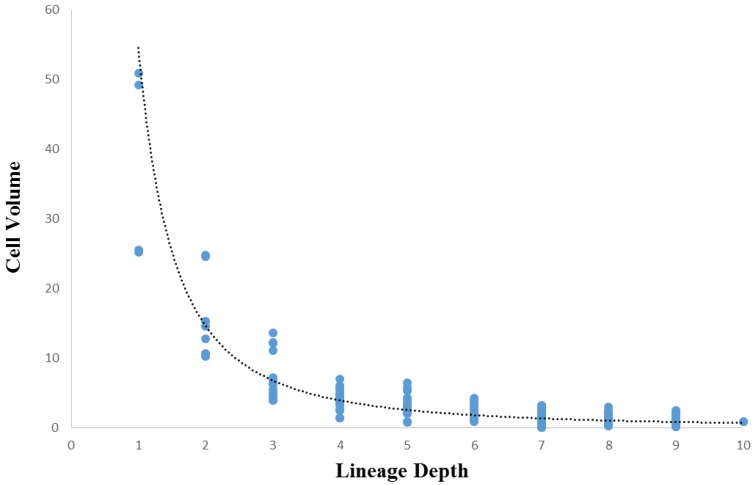
Comparison of cell volume versus lineage depth for *Ciona*. The graph includes 382 cells from the two-cell stage to the 112-cell stage. Lineage depth is determined by the number of division events from the one-cell stage (depth of 0).

**Figure 8:**
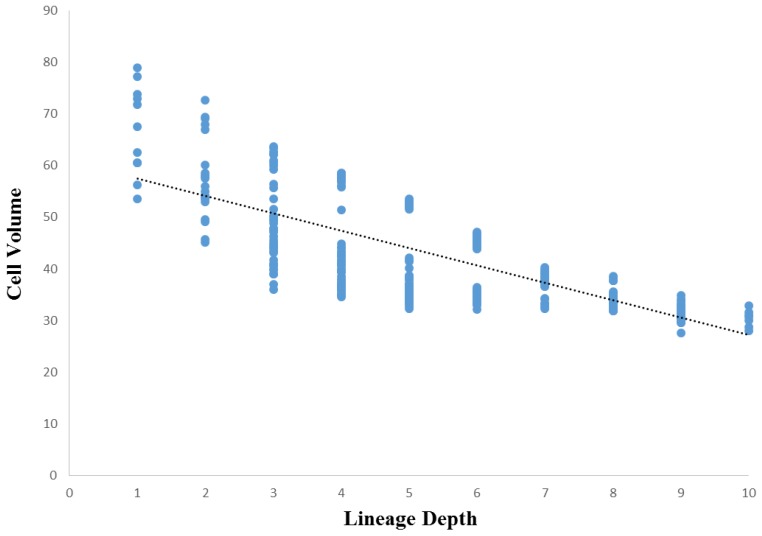
Comparison of relative cell volume versus lineage depth for *C. elegans*. Graph includes 558 cells from the pre-hatch embryo. Lineage depth is determined by the number of division events from the one-cell stage (depth of 0).

Comparisons between cell volume and lineage depth are shown for *Ciona* in Figure 7 and *C. elegans* in Figure 8. In *Ciona*, the relationship between lineage depth and cell volume can be fitted to a power function (see Methods, Section 2.8), *R*^2^ = 0.60, *p* < 0.001. An identical bivariate comparison in *C. elegans* yields a linear regression of similar strength (*R*^2^ = 0.56, *p* < 0.001). In both cases, we can see that overall, cell volume decreases as lineage depth increases, but it is noteworthy that the curve is nonlinear for *Ciona* (Figure 7) while linear for *C. elegans* (Figure 8). This is consistent with the decrease in volume over developmental time that was shown in Figure 5 (*Ciona*) and Figure 6 (*C. elegans*).

Since there are a multitude of volume above-threshold asymmetric binary cell divisions in each dataset, we will now turn our attention to the volume and lineage depth relationship in the context of these types of divisions. We can then ask whether these volume asymmetries are large enough across the embryo to create a different pattern for smaller cells and larger cells in individual divisions. This is particularly relevant in light of the order-of-magnitude difference in above-threshold asymmetric binary divisions (Table 1 and Table 2).

**Figure 9:**
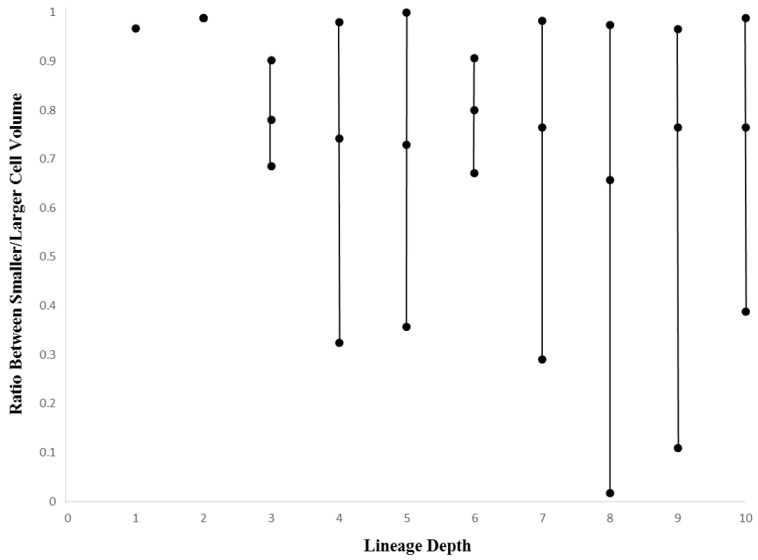
Maximum, mean, and minimum values (black dots from top to bottom) for smaller cell volume to larger cell volume ratios for *Ciona* summarized by lineage depth (values per division event).

**Figure 10:**
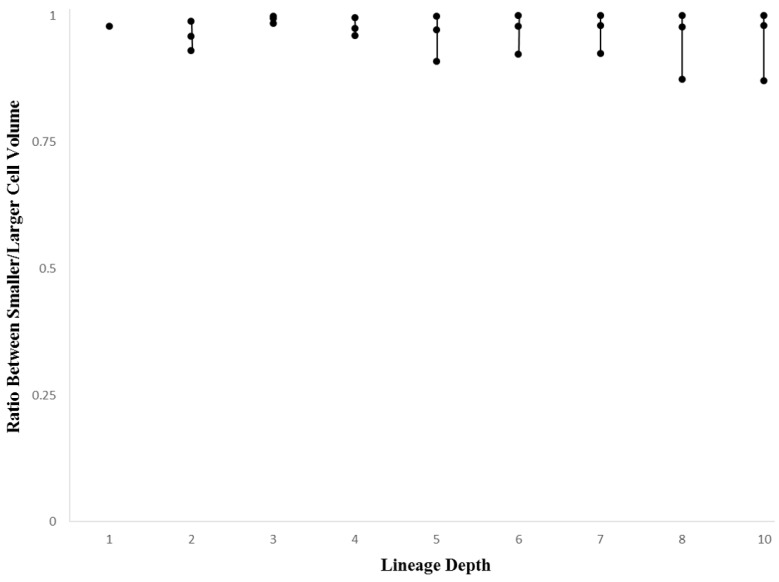
Maximum, mean, and minimum values (black dots from top to bottom) for smaller cell volume to larger cell volume ratios for *C. elegans* summarized by lineage depth (values per division event).

To examine the variation in volume across pairs of daughter cells, we can look at the volumetric ratio between the smaller and larger daughter cell (see Methods, Section 2.5). This ratio can be stratified in terms of depth in a single lineage, while also being comparable more generally across species. Such a measure gives us a global heuristic in terms of detecting the relative contribution of asymmetrical divisions at different depths of a given lineage tree. Figure 9 (*Ciona*) and Figure 10 (*C. elegans*) show the range (minimum, mean, and maximum values) of cell volume ratios. For both species, there is a tendency for greater asymmetry as the number of division events increase (e.g., an increase in lineage depth). For the first few depths of the lineage tree (up to the eight-cell stage in *Ciona* and 16-cell stage in *C. elegans*), there are virtually no variations in cell asymmetry, and the ratio between smaller cell to larger cell is very close to one. It is of note that the scale of variation is much larger in the *Ciona* embryo, perhaps reflecting a combination of aforementioned technical variation and the nature of differentiation as captured by the lineage tree. Variation is observed only later in the lineage tree, which may represent selective differentiation events in different parts of the tree. While the mean value decreases in *Ciona* after this point (and does not decrease in *C. elegans*), the value fluctuates rather than decreasing monotonically. This may point to a non-uniform but consistent number of cell divisions that exhibit above-threshold volumetric asymmetry.

### 3.4. Comparison of Lineage and Differentiation Trees

While there appear to be similarities in cell volume with respect to lineage depth, we still do not know what information asymmetries in cell volume tell us about the bifurcation process. Since both *Ciona* and *C. elegans* demonstrate binary divisions as a default mode of differentiation, we can investigate whether or not the difference between the differentiation code and the lineage code can capture major ordering differences between the lineage tree and differentiation tree. If this is indeed the case, the volume differences shown previously could be restricted to specific subtrees, and perhaps indicate functional significance that has its origins at specific points in the lineage tree and locations in the embryo. A list of lineage codes and differentiation codes are available in Table S4 for *Ciona* and Table S5 for *C. elegans*.

**Figure 11:**
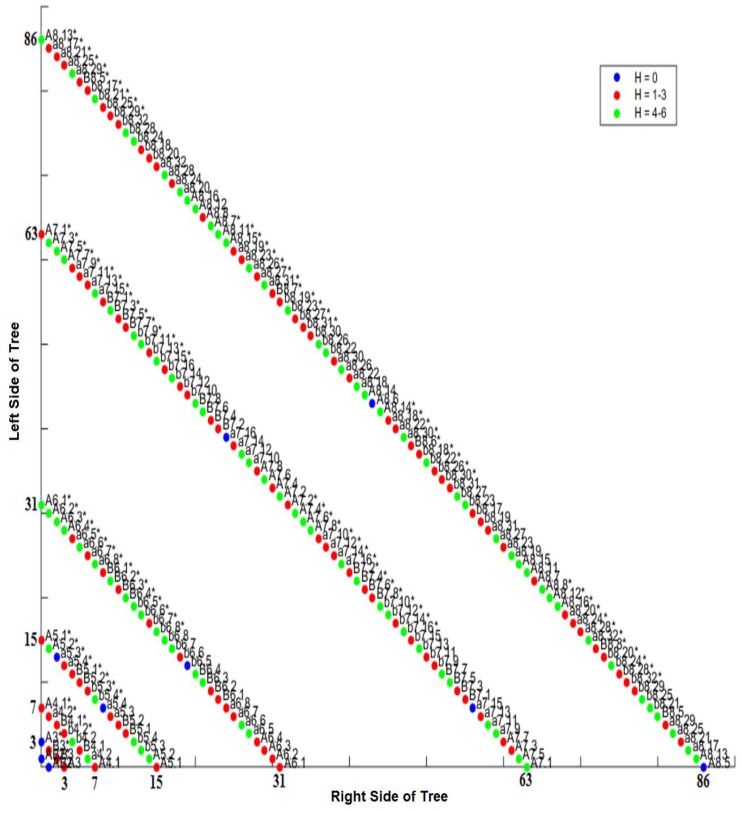
An isometric graph showing the Hamming distance of the differentiation tree from the lineage tree in *Ciona* (N = 213). The *H* abbreviation stands for Hamming distance. The position of a point representing a cell is based on the depth of its node in the differentiation tree. The positions of all points are rotated 45 degrees clockwise from a bottom-to-top differentiation tree ordering (where the 1-cell stage is at the bottom of the graph). Each cell is colored with its Hamming distance. Along a given line, the cells appear in their order, left to right, in the differentiation tree. For example, cells A.5.1 and A.5.1* at depth 4, where the total number of cells is 16 (ranging from 0 to 15), means cell A.5.1 is at the extreme left side of the differentiation tree, and cell A.5.1* is at the extreme right side of the differentiation tree. The other cells are spaced in between. The pattern of Hamming distances suggests that the relationship between the two orderings is nonrandom and therefore has some underlying anatomical importance.

One way we can compare the lineage tree order and the differentiation tree order is by calculating a numeric distance between the differentiation code and the lineage code. Since these binary codes provide specific addresses for each cell in the embryo, specific cells (and locations in the tree) can be compared between tree orderings, quantified using the Hamming distance. This distance between the lineage code and differentiation code is measured in bits (see Methods, Section 2.9). We use the lineage tree to root the lineage code/differentiation code comparison in *Ciona* and the differentiation tree to do the same in *C. elegans,* to demonstrate that both can be effectively used to determine the Hamming distance for individual cells. This makes no difference in terms of the Hamming distance for each cell nor the mother cell/descendant relationship. However, the lineage tree for *Ciona* is used because the *Ciona* differentiation tree potentially contains many more potential errors than the lineage tree. Measurable differences between the lineage code and differentiation code using the Hamming distance metric were visualized using an isometric graph (Figure 11 for *Ciona* and Figure 11 for *C. elegans*).

### 3.5. Intra-Specific Comparisons Using Isometric Graphs

The isometric graph considers the lineage tree as a so-called depth tree rather than a real-time tree. In a depth tree, the interval from one cell division to another is marked by discrete intervals which are analogous to the division events in the differentiation trees shown in Figures S1 (*Ciona*) and S2 (*C. elegans*). An isometric graph projects a layered differentiation tree/lineage tree onto a bivariate space at a counter-clockwise rotation of = 135 degrees. This provides us with the depths of the tree without showing the edges. The root (one-cell node) is at the lower left-hand corner (0,0) of the plot. Composed of a series of isometric lines, isometric graphs allow us to convert the differentiation code to an (*m,n*) pair, where m is the depth of a given node and n is a number referring to order of the cell along the line for depth *m*.

**Figure 12:**
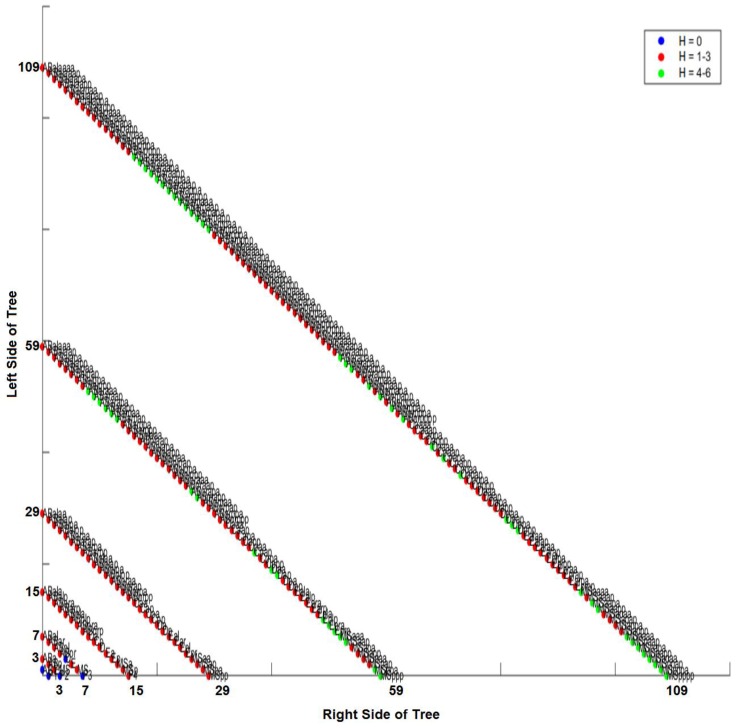
An isometric graph showing the Hamming distance of the differentiation tree from the lineage tree in *C. elegans* (N = 230). The *H* abbreviation stands for Hamming distance. The position of a point representing a cell is based on the depth of its node in the differentiation tree. The positions of all points are rotated 45 degrees clockwise from a bottom-to-top differentiation tree ordering (where the one-cell stage is at the bottom of the graph). Each cell is colored with its Hamming distance. See the legend of Figure 11 for other details.

Each depth of the tree is represented by a discrete isometric line. These isometric lines are drawn diagonally across the graph, and represent the number of cells at each depth of the tree. The numbers on the axes of both Figure 11 and Figure 12 represent the length (number of cells) in a given depth of the lineage tree. For example, in Figure 11, the fourth depth (lineage depth of 4) in the *Ciona* tree has a total of 32 cells, and crosses both the x- and y-axes at 31 (with cells at that level numbered from 0 to 31). This type of contour map allows us to overlay the differentiation and lineage tree orders, as well as to visualize both the isomorphic and non-isomorphic components of this overlap.

One notable outcome in both Figure 11 and Figure 12 is that a change in the node ordering and Hamming distance due to a change in classification criterion can affect all of the descendent cells of a given cell, which can be referred to as the cell’s sub-tree. If this change occurs at a shallow depth in the tree (e.g., at eight-cell stage), this may produce proportionally large subtrees (as compared with the entire tree). We can see an example of Hamming distance variation amongst descendent cells in the *Ciona* isometric graph (Figure 11) by looking at cells A6.1 and A6.2 (depth 5, isoline of value 31) in the lineage tree. Both of these cells are 1–3 bits from their differentiation code. The descendants of A6.1 at depth 6 (isoline of value 63) are 4–6 bits different, while the descendants of A6.2 at depth 6 remain 1–3 bits different. It is at depth 7 where we begin to see a significant degree of variation: the Hamming distance for A6.1 descendants at depth 8 (isoline of value 86) range from 0 bits (25%) to 4–6 bits (50%). The descendants of A6.2 exhibit Hamming distances ranging from 1–3 bits (25%) to 4–6 bits (75%). Conversely, more uniform changes can define entire sub-lineages. By using the composite differentiation code (see Methods) instead of the comparison shown for *C. elegans* in Figure 12, all cells in the P1 sub-lineage will be an additional bit away from the lineage tree as compared to the AB sub-lineage. Figure 12 provides another example of uniform changes of Hamming distance in the *C. elegans* isometric graph by looking at cell MSp, which is the first posterior (p) daughter of the MS sublineage founder cell, where M and S are arbitrary labels for this founder cell; depth 4, isoline value of 15. MSp has a Hamming distance of 1–3 bits from its lineage code. Similarly, the immediate descendants of MSp (depth 5, isoline value of 29) also have a Hamming distance of 1–3 bits. The descendants of MS at depth 6 (isoline value of 59) have values based on their mothers at depth 5: the daughters of MSpa retain a Hamming distance of of 1–3 bits (50%), while the daughters of MSpp exhibit a value of 4–6 bits (50%). At depth 7 (isoline value of 109), all descendants of MSp (100%) have a Hamming distance of 4–6 bits. Another way to distinguish Hamming distance distributions in subtrees and single tree depths from random noise is to compare randomly-generated trees with those from known organisms (e.g., *C. elegans*). When this comparison is made (see [72]), a biological signal is defined by a level-specific uniformity of Hamming distance values within subtrees as illustrated above.

An additional question involves the existence of meaningful spatial variation amongst these Hamming distances. Using *C. elegans* as an example, a 4-dimensional plot (3 dimensions of space and one of binary distance) was constructed (Figure S4). While there are no clearly separable spatial patterns, strings of cells along the three anatomical axes all with the same Hamming distance do exist (e.g., points with a Hamming distance of 2 along the A-P axis). At least some of this variation is due to constituents of the same sub-tree and with the same Hamming distance in spatial locations being in positions described by their nomenclature.

### 3.6. CAST Analysis of Differentiation Codes

**Figure 13:**
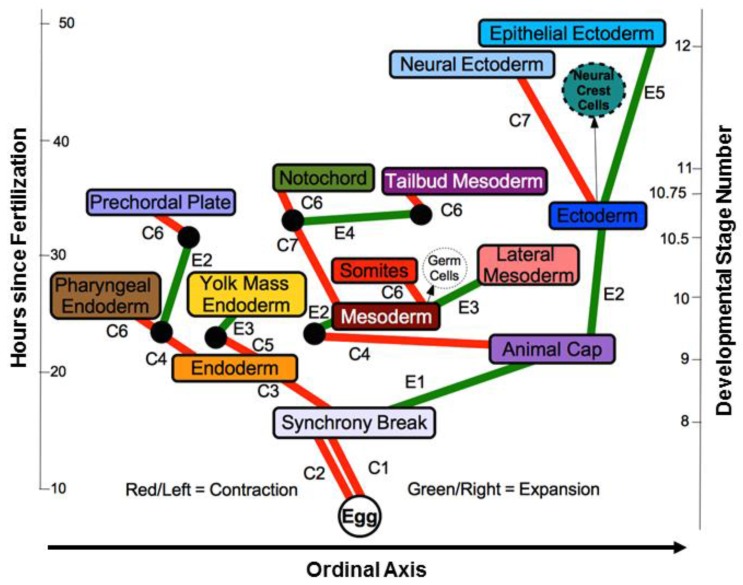
The differentiation tree of the axolotl (*Ambystoma mexicanum*) up to neural plate formation (stages 8–12 raised at 20 °C). All cell types are presumptive. Red branches to the left labelled with the letter C indicates a contraction wave, while green branches to the right labelled with the letter E indicates an expansion wave. The time axis (vertical) is represented in two ways: (1) on the left, are hours since fertilization for embryos raised at 20 °C, (2) on the right are stage numbers based on distinctly recognizable morphological features [49]. Regarded as a vector, the vertical component of each edge represents the duration of each wave. The ordinal axis is horizontal in this figure. Note each numbered wave may pass through one tissue and continue into an adjacent tissue and so, for example, E3 begins in endodermal tissue and then continues into mesodermal tissue. From [10] with permission of World Scientific Publishing. The differentiation codes based on the named tissues (boxes) and unnamed intermediate tissues (black dots) with unique differentiation codes are determined from this tree and shown in Table S6.

Another analysis of the data involves transforming the differentiation code into a series of letters and then aligning them to achieve a comparison between species or mutant genotypes within a species. The CAST codes for *Ciona* and *C. elegans* are located in Tables S4 and 5, respectively. Our convention (see Methods) is already in use for the differentiation tree of *Ambystoma mexicanum* (Urodele Salamander, axolotl). This differentiation tree is shown in Figure 13. In mosaic development, a smaller cell in a binary division represents a contraction wave, and a larger cell in a binary division represents an expansion wave (see [13]). The differentiation codes classify single differentiation trees, while the CAST translation allows for alignment with other differentiation trees to find common motifs or alignment gaps. For example, we might want to discover repeatable sequences of differentiations across species. Through comparison with lineage trees and fate maps, CAST code alignments and motif finding might uncover how portions of the differentiation tree topology get duplicated across evolution. In particular, CASTing allows a comparison between the differentiation trees of mosaic and regulating embryos.

In the example given in Table S6, we align the CAST codes for *Ciona* and *C. elegans*. The alignment is done by matching identical sequences, and leaving a gap of a certain length to between exact matches. Using a variation on dynamic programming [71], a score is calculated to evaluate the alignment. The *Ciona*/*C. elegans* alignment revealed a length of 257 and a score of 128 (50% of the length). A *Ciona*/*C. elegans* alignment conducted with the composite differentiation code (see Methods) converted into a CAST code for *C. elegans* revealed the same length (257) and a score of 120 (47% of the length). While this score points to significant differences in the differentiation tree structure between the two species, it also indicates the reliability of using motif finding techniques to compare two or more differentiation trees.

Although we only provide a demonstration of the technique, CAST codes complement the Hamming distance analysis by focusing on variation across differentiation trees. In a number of *C. elegans* mutant genotypes [73,74], single allele mutations can target the developmental process and disrupt both the timing and topology of lineage trees. Of course, this can also produce changes in the differentiation tree by disrupting embryonic organization at a global scale [75].

After generating the differentiation code for the axolotl tree and converting it to a CAST code, we then aligned the CAST code for two chordate species with different types of development: *Ciona* (mosaic development) and axolotl (regulative development). This alignment is shown in Table S6. From the results of this alignment, we can identify a major obstacle to aligning CAST codes from mosaic and regulative species. A full alignment, including all cells in *Ciona* and all tissues in axolotl, gives us a length of 209 and a score of −177, due to the large number of gaps. However, shortening the alignment to include only the first three depths of each differentiation tree as represented by the CAST code (e.g., A., B., and C., in Table S6) gives us a length of 14 and a score of 6 (43% of the length). A similarly truncated alignment of *C. elegans* and axolotl, conducted using both the regular and composite CAST codes, yields a length of 14 and a score of 6 (43% of the length). These results are not surprising, as there are fewer tissue types in a complex regulative organism at early stages than cells in a complex mosaic organism. Thus using the first few depths of a CAST code derived from a mosaic organism allows us to make comparisons between founder cells of the mosaic tree and tissue types of the regulative tree.

### 3.7. Interpretation of Results

The examination of within species cellular variation is done to demonstrate the degree of commonality between taxa with different evolutionary histories, which might provide clues to parallel evolutionary processes. As we can see in the results, there are indeed commonalities between *C. elegans* and *Ciona* that may deserve further study. One of these is the existence of two categories of unequal size for comparisons of cell lifetime versus volume. Related to this is the existence of linear to power law scaling for the relationship between cell division time versus volume. Taken together, these findings suggest a set of common mechanisms that are perhaps integral to the embryogenetic process. These findings are also consistent with previous studies [42,45], which serves to reinforce our confidence in the comparison.

A more general investigation of cellular variation is done to reinforce the degree of commonality between taxa with different evolutionary histories in addition to establishing the role of evolutionary conservation in the dynamics of the mosaic differentiation process. While the more general investigation of cellular variation yielded somewhat ambiguous results, there is reason to believe that these results also show similarities between *Ciona* and *C. elegans* mosaic development. Although the cell volume versus lineage depth graphs for *Ciona* and *C. elegans* (Figure 7 and Figure 8, respectively) demonstrates differences in how cell size decreases with respect to lineage tree depth, we also observe differences in the cell size ratio between *Ciona* and *C. elegans* (Figure 9 and Figure 10, respectively). These variations stem from the different ways in which we estimate volume (nuclei segmentation versus segmentation of membranes in embryo). However, we also know that in *C. elegans*, the relationship between cell size and cell nuclei tends to conform to a power function [45], with the largest cells (e.g., first two divisions in lineage tree) being much larger than their corresponding nuclei. If we take this relationship between nucleus and membrane-delimited cell volume into account, we can interpret the cell volume versus lineage depth graphs for *C. elegans* as being very similar to *Ciona*.

The various means of constructing and analyzing differentiation trees provide ways to examine differences in the mosaic developmental process between taxa with different evolutionary histories. In the case of the CAST code analysis, we include an exemplar regulative taxon (axolotl) to demonstrate the generalizability of our approach. In the case of the isometric graph approach, the differentiation trees for *Ciona* and *C. elegans* (Figure 11 and Figure 12, respectively) reveal a non-random signal [72] that might be useful to understanding the conservation of sub-tree topologies and other features of mosaic development. For the CAST code analysis, we can see that while there are major differences in differentiation trees between mosaic and regulative systems, there are also smaller-scale differences between mosaic systems with different evolutionary histories.

## 4. Discussion

There are several implications for this work that may inform our understanding of the mosaic developmental process across evolutionary contexts. This work goes beyond the typical focus on homology and phylogeny, and focuses instead on the necessary functional features of a deterministic developmental system. In this sense, we are establishing a means for studying evo-embryo-devo. While we recognize that mosaic developmental mechanisms may very well be a product of parallel evolution, there are several universal features, each of which are likely to characterize most instances of mosaic development in the tree of life. In terms of establishing a common set of features across biological contexts, this is similar to the study of flight from a biomechanical perspective [76]. On the other hand, commonalities might be inherited from a common ancestor.

### 4.1. Analytical Caveats

Our study comes with multiple caveats that may reduce the usefulness of our comparison. One of these caveats involves an uneven comparison of developmental context. A major caveat is the nature of the available data, particularly using the GFP^+^ nuclei to estimate cell volume in *C. elegans*. While the datasets used are comparable in some ways, they do not serve us particularly well in others. This limitation is particularly true for the lack of size variation amongst *C. elegans* daughter cell pairs. Our estimates for smaller and larger divisions would be improved by data on whole cell size, or cell volume data that resembles the method of segmentation for *Ciona*. For example, our method of estimating *C. elegans* cell size may misclassify some pairs of cells by not capturing the true extent of their shape and size. This is particularly true of cell polarity exhibited during the two and four-cell stage in *C. elegans* [64]. It is of note that there would be a similar issue with defining size by segmenting the cell membrane, as the complexities of cell shape are also hard to capture in an absolute manner.

Another caveat involves the organization and nomenclature of existing lineage trees for both *C. elegans* and *Ciona*. Some of this organization indeed reflects the uniqueness of each species’ development and biology, but also involves technical variation. In the case of comparisons between lineage and differentiation trees, it is important to realize that differences in ordering reflect potential differences at many different levels of biological complexity. Using *C. elegans* as an example, differences in volume or axial position amongst daughter cells may be due to gene expression differences, ultrastructural differences, and/or simply unexplored stochastic processes. Disentangling these sources of variation is a subject for future research. Finally, there may be alternative methods to reorder the lineage tree in a way that reveals additional information about differentiation waves. One way to do this reordering is to use complexity measures for lineage trees that allow us to take into account various sub-tree relationships and evolutionary constraints [77].

### 4.2. Discussion of Within-Species Cellular Variation

Beyond the scope of these analyses, there are sources of variation in mosaic developmental mechanisms that contribute to statistical differences observed between these species. We have confirmed that *C. elegans* exhibits autonomous specification of its cells in development. However, in the case of certain specialized functions a variety of mechanisms supplement this autonomy, and is in some ways similar to species that exhibit regulative development [78]. For example, cell cycle duration can be contingent upon lineage-specificity in *C. elegans*. This seems to be the case as early as the two-cell embryo [79]. In this example, the founder cell of the AB sub-lineage (AB) takes less time to divide than the P1 cell [79], which may also explain the differential presence of outliers between *Ciona* (Figure 5 and Figure 7) and *C. elegans* (Figure 6 and Figure 8). In the case of *Ciona* (Figure 5 and Figure 7), deviation from a linear trend before the eight-cell stage (captured as a power function) may largely be due to the way in which cell volume is measured. However, the role of cellular differentiation in the formation of four spatially-localized blastomeres previous to the eight-cell stage might also play a role. As in the case of the founder cell of the germ cell sub-lineage (P1) in *C. elegans* [80], these eight cells serve a particular biological function not directly involved with morphogenesis or differentiation into tissue types.

### 4.3. Differentiation Trees and Comparative Development

By going beyond the standard lineage tree representation of early development [81], we can begin to uncover information about the tempo, mode, and universals of mosaic embryogenesis. However, it is still an open question as to whether many of our findings reveal the precursors of structural and functional similarities and/or differences observed in adulthood [82]. We do know that characterization of asymmetric divisions in the embryo can tell us something about the establishment of differentiation well before the development and maturation of tissue types [13,32]. For example, even though *C. elegans* embryos reveal less cell volume asymmetry on average upon division (see Table S3), we observe larger differences between sister cells in the P1 sublineage. This should be expected, as this sub-lineage contains a greater diversity of founder cells for highly-specialized lineages (e.g., D, C, E, and MS) than the AB sub-lineage.

Cell divisions in *Ciona* occur at different depths and exhibit variation due to a number of factors. One driving force behind this variation is that there is a finite number of divisions in embryonic cells before differentiation into tissues [83]. In both *C. elegans* and *Ciona*, asymmetrical cell divisions are expected to occur as early as the eight-cell stage. In *C. elegans*, early asymmetric divisions (e.g., the first few divisions after P0) are essential for determining anatomical axes [84]. In *Ciona*, divisions which are unequal in size can be specific to spatial location (e.g., posterior blastomeres), and prepare the embryo for convergent extension during gastrulation [85]. For example, the sublineage founded by the B4.1 pair of blastomeres features a series of three sequential asymmetric divisions. Each of these asymmetric divisions are spatially-oriented (e.g., toward the posterior end) [85].

It has been suggested in [13] that for mosaic organisms, highly asymmetric divisions that lead to cellular differentiation stand in contrast to symmetric divisions that lead to cell proliferation. We can also observe this in the asymmetric mode of division within different subtrees. This is particularly true in the case of *Ciona* (see Table S2), which presents us with four-fold symmetry and a fairly large volume disparity in binary divisions at the 76-cell and 112-cell depths of the differentiation tree (see Figure S1A, Figure S1B, and Table S2). While the four-fold body plan is evolutionarily-distinct from the bilateral symmetry of *C. elegans* [86], CASTing suggests there are commonalities waiting to be discovered.

In the theory of differentiation waves, it is also proposed that natural selection works to synchronize cell state splitters via differentiation waves if there are selective pressures for larger tissue volumes [13,87]. Given the eutelic nature of both *Ciona* and *C. elegans*, we should be able to see evidence of this link between the synchronization of binary divisions and the production of similar cell types. Indeed, we observe both differences in the timing of binary divisions between the AB and P1 sublineages in *C. elegans* [65] and the blastomeres of the 8-cell *Ciona* embryo [75]. Whether these timing differences correspond to later functional differences or embryonic position is an interesting question for future studies.

### 4.4. Overarching Features of Mosaic Embryogenesis

There are three main takeaways from our cross-species study of mosaic embryogenesis. The first is that both linear and nonlinear functions [88,89] can be used to define the nature of differentiation events over time in a specific organismal context. In this case, statistical scaling (e.g. biological processes described by a mathematical function) characterizes generalized differentiation events in an organism over developmental time, such as observed in Figure 5 for *Ciona* and Figure 4 and Figure 6 for *C. elegans*. Envision this as the unfolding of an initial condition. The initial developmental condition, in this case a 1-cell embryo with a genetic program, requires a developmental process to shape and partition this initial single cell. In the case of the mosaic developmental process, our one-cell embryo requires a basic set of invariant rules in order to produce a differentiated embryo of reasonable fitness. Interpreting our findings as a series of scaling laws suggest that changes in cell volume over time may be concurrent with the many other dynamic parameters (e.g., metabolic, chemical) found in a complex organism [89].

We also propose that there are there are two universal parameters of the differentiation process: the initial volume of a founder cell (e.g., first cell of a sub-lineage, one example being AB in *C. elegans*), and the scale of cell volume differences between daughter cells. This follows from the one-cell embryo example: the initial volume of the one-cell embryo will determine the number of divisions and/or the volume range of descendent cells. Given our analysis of the *Ciona* embryo, it appears that large volume asymmetries during the developmental process are restricted to either specific events or relatively small subtrees. Whether specific instances of volume asymmetry are due to stochastic processes or functional processes is beyond the scope of this study.

We also propose that the distance between lineage and differentiation tree topologies based on different classificatory criteria reveals locations in the lineage tree that may have cryptic functional significance. More specifically, this may enable the embryo to prepare for higher-order differentiation events such as tissue formation. Our comparison enables a superficial examination of the interaction between axial position (lineage tree) and multiple levels of categorization by volume (differentiation tree). This examination allows us to refine general trends of volume reduction in descendent cells by axial location, three-dimensional spatial position, or other localized contexts, and gives us the ability to put differences in scaling between species into these localized contexts.

### 4.5. Broader Evolutionary Implications

We can also look to the evolution of development (evo-devo) literature to gain perspective on how this type of cross-species analysis can inform our understanding of the influence of evolutionary processes on development. Given different evolutionary histories, we are nevertheless able to identify specific, possibly universal, developmental features through conversion of lineage trees to differentiation trees, the graphical analysis of lineage and differentiation codes, alignment of CAST codes from different species, and comparing the scaling of division time to cell volume across species. One possible explanation for consistencies in the developmental process across species is the coupling of developmental modularity and evolutionary constraints [90]. In the case of *Ciona* and *C. elegans*, we may be observing the expression of tightly-constrained developmental modules related to mosaic development.

Even in cases of parallel evolution, the evolution of specific morphologies rely upon the underlying coordination of embryogenesis in both space and time [91]. In this way, developmental modularity also allows us to make the association between morphological units and developmental processes [90]. Comparing broadly across the tree of life provides us with some insight into how these constraints operate on and otherwise coordinate developmental processes in different biological contexts. Future work will include converting the features of a differentiation tree, particularly the structure of sub-trees, into characters suitable for phylogeny reconstruction, which should provide much-needed developmental context for the inference of evolutionary relationships.

### 4.6. A Toy Model of an Idealized Mosaic Embryo

In terms of comparing within and between mosaic developing species, why do we ultimately care about such an abstract set of properties? This type of approach provides us with a toy model [92] that perhaps allows us to generate the properties of a generic mosaic embryo. The approach is similar to that of a hypothetical common ancestor [93], except that there is no assumption of monophyly (common ancestry). While various mosaic-developing organisms may execute their program in different ways, we believe that the overarching structure of mosaic development can be distilled to its essential features. By utilizing the theory and methods of differentiation trees and codes, we have taken the first step in this direction.

There are a number of reasons for preferring an overarching model of mosaic embryogenesis over a highly-contingent one. One advantage is that it might enable the creation of generic, bootable organisms [94,95]. While this is generally accomplished using microbial life, we could also create analogues for chordates and invertebrates. In the case of *C. elegans*, initiatives such as OpenWorm [96,97] might provide the tools for booting up the adult form of this organism. Finally, the differentiation tree approach and assorted tools might help us to understand the structure and global properties of developmental cell lineages that lineage trees, fate maps, and high-resolution microscopy simply cannot do on their own. This is of course useful in creating life, but even more so for understanding it.

## 5. Conclusions

In [13], it is proposed that differentiation trees might offer the best criteria for construction of phylogenetic trees. This paper is the first step in that direction, showing that even between widely disparate animals there are hints of commonalities in their differentiation trees, based on quantitative criteria for comparing them. Beyond the ordinary graph, every edge of a differentiation tree has unique properties. Thus molecular phylogenies should be nested within and thereby complement phylogenies based on differentiation trees. Future work might include integrating the comparative study of embryology more tightly into the modern synthesis using the techniques that we have introduced in this paper.

In this paper, we have taken a strictly graph theoretical approach to comparing lineage and differentiation trees, reducing cell differentiation to a sequence of 0’s and 1’s. This is, of course, a simplification of the process, as different kinds of cells are generated at each step. In future work, we will consider whether tagging cells with their actual or presumptive roles (based on fate maps) will improve CASTing scores.

We have chosen three organisms here that are far-flung from one another on the tree of life. That we find hints of interesting parallels between their differentiation trees, and perhaps homologies, is encouraging. For future work we will investigate the relationships between the differentiation trees of very closely related organisms, such as between species of nematodes [98,99] and between mutants of *C. elegans* [28]. Here, of course, we anticipate high CASTing scores, but, more importantly, insights into evolutionary mechanisms at the level of cell differentiation.

With the vast increase in our ability to track all of the cells in developing embryos, both mosaic and regulating, we hope that our work will provide incentives to ascertain not only cell trajectories, but differentiation events, quantification of asymmetric cleavages, and recording of differentiation waves, across many species. It would be far better that investigators deliberately record these parameters as best as possible, than to have to eke them out of databases the way we have done so here.

That both modes of development, mosaic and regulative, must have a common basis is obvious, because both are found in each phylogenetic group. The concept of differentiation trees provides the first unifying theory for mosaic and regulative development, and for their evolution. Our work contributes to efforts that go beyond a Modern Synthesis-influenced view that emphasizes population dynamics and Mendelian genetics [33]. This includes numerous parallels with the literature on the emerging Postmodern Synthesis, including understanding embryogenetic dynamics from an evolutionary perspective [33] and the role of biological form and functional morphology in the evolutionary process [34].

## Figures and Tables

**Table 1:** Number and proportion of cell divisions above confidence interval threshold (see Methods) for asymmetric cell divisions, based on Equation (2). Data represents all divisions (N = 117) in a pre-gastrulation *Ciona* fate lineage tree.

*Ciona*	Confidence Interval h				
	0.05	0.1	0.25	0.50	TOTAL
Number of volume asymmetric cell divisions	103	82	48	23	117
Proportion of total	0.88	0.7	0.41	0.2	1.0

**Table 2:** Number and proportion of cell divisions above confidence interval threshold (see Methods) for asymmetric cell divisions. Data represents all divisions (N = 257) in a pre-hatch *C. elegans* lineage tree.

*C. elegans*	Confidence Interval h				
	0.005	0.01	0.025	0.05	TOTAL
Number of volume asymmetric cell divisions	203	151	80	22	257
Proportion of total	0.79	0.59	0.31	0.09	1.0

## Supplementary Materials

The following are available online: www.mdpi.com/2079-7737/5/3/33/s1. Figure S1A: *Ciona* differentiation tree ordering, 10 division events. Differentiation tree only (no tissue precursor definitions shown); Figure S1B: *Ciona* differentiation tree ordering, 10 division events. Differentiation tree with tissue precursor definitions color coded; Figure S2A: *C. elegans* differentiation tree ordering, 8 division events. Differentiation tree only (no tissue precursor definitions shown); Figure S2B: *C. elegans* differentiation tree ordering, 8 division events. Differentiation tree with tissue precursor definitions color coded; Figure S3: Bivariate graph of division time versus lifetime of cell for *Ciona* and *C. elegans*; Figure S4: A three-dimensional plot of cell positions in the *C. elegans* embryo showing Hamming distances between the lineage and differentiation tree for three-dimensional cell positions. This corresponds to nine division events for the differentiation tree in Figure s2A and Figure S2B; Table S1: Embryo volume per cell stage of embryogenesis for *Ciona*; Table S2: *Ciona* confidence intervals (CIs); Table S3: *C. elegans* confidence intervals (CIs); Table S4: Excel spreadsheets, of *Ciona* lineage code, *Ciona* differentiation code, *Ciona* CAST code, and frequency counts and probabilities for Hamming distances per subtree; Table S5: Excel spreadsheets, of *C. elegans* lineage code, *C. elegans* differentiation code, *C. elegans* composite differentiation code, *C. elegans* CAST code, and frequency counts and probabilities for Hamming distances per subtree; Table S6: Excel spreadsheet that demonstrates the alignment and alignment score of CAST codes for *C. elegans* and *Ciona* differentiation trees, and the alignment and alignment score of CAST codes for axolotl and *Ciona* differentiation trees.
