# Supplementary material for: Quantifying Mosaic Development: Towards an Evo-Devo Postmodern Synthesis of the Evolution of Development via Differentiation Trees of Embryos
Source: Biology (Basel). 2016 Aug 18;5(3):33. doi: 10.3390/biology5030033 (PMC5037352; doi:10.3390/biology5030033)
Supplement: Supplementary file 1 [file biology-05-00033-s001.zip › biology-127827-supplementry - final/Table S1.docx]

**Table S1.** Embryo volume per cell stage of embryogenesis for *Ciona*. Volume (measured in cubic microns) is averaged over a number of observations (N). Data is graphed in Figure 1.

| **Cell Stage** | **N** | **Volume (µm^3^)** |
| --- | --- | --- |
| 2 | 1 | 2136600 |
| 4 | 1 | 2819884 |
| 8 | 2 | 2316572 |
| 16 | 4 | 1747595 |
| 24 | 1 | 1771553 |
| 32 | 13 | 1648322 |
| 44 | 4 | 1989783 |
| 64 | 3 | 1456609 |
| 76 | 3 | 2572317 |
| 112 | 6 | 1965414 |
