# Supplementary figures and images for: Quantifying Mosaic Development: Towards an Evo-Devo Postmodern Synthesis of the Evolution of Development via Differentiation Trees of Embryos
Source: Biology (Basel). 2016 Aug 18;5(3):33. doi: 10.3390/biology5030033 (PMC5037352; doi:10.3390/biology5030033)

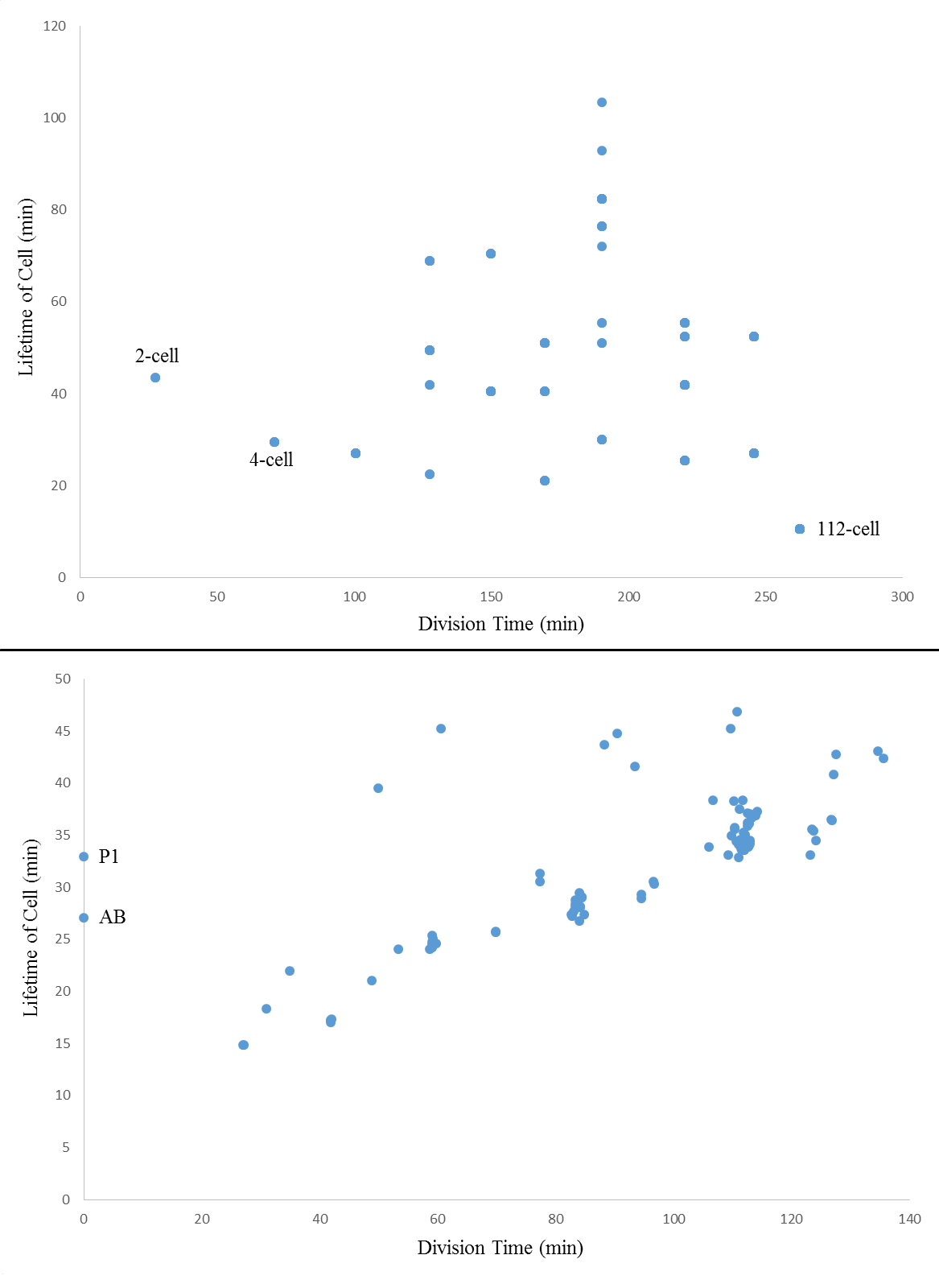

Supplement: Supplementary file 1 [file biology-05-00033-s001.zip › biology-127827-supplementry - final/Fig S3.png]

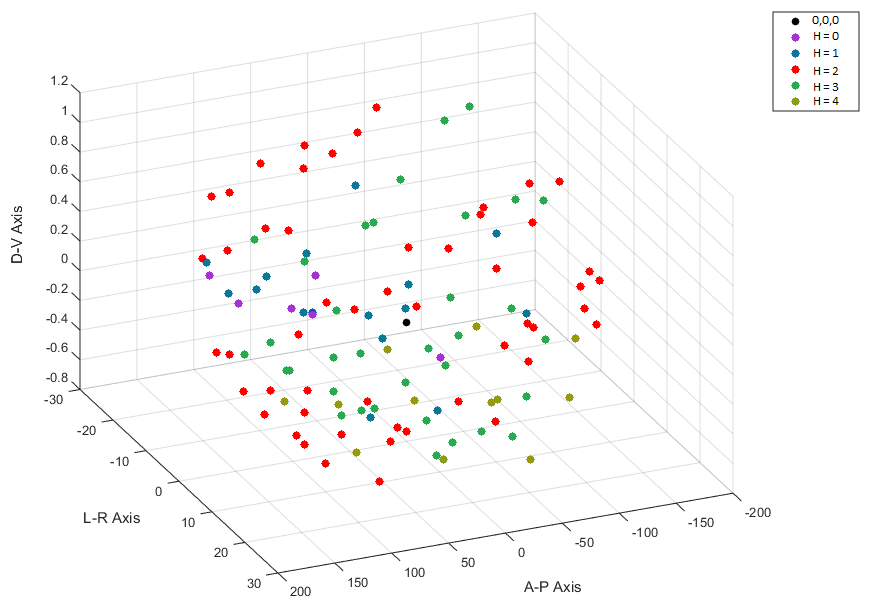

Supplement: Supplementary file 1 [file biology-05-00033-s001.zip › biology-127827-supplementry - final/Fig S4.png]
